# Efficacy and safety of netupitant/palonosetron in preventing nausea and vomiting in diffuse large B cell lymphoma patients undergoing R–CHOP chemotherapy

**DOI:** 10.1038/s41598-024-62057-4

**Published:** 2024-05-16

**Authors:** Kunye Kwak, Yong Park, Byung Soo Kim, Ka-Won Kang

**Affiliations:** Division of Hematology-Oncology, Department of Internal Medicine, Korea University College of Medicine, 73, Goryeodae-Ro, Seongbuk-Gu, Seoul, 02841 Republic of Korea

**Keywords:** Chemotherapy induced nausea and vomiting, Netupitant, Palonosetron, Diffuse large B cell lymphoma, Chemotherapy, Antiemetics, Cancer, Quality of life

## Abstract

Diffuse large B-cell lymphoma (DLBCL) is the most common type of non-Hodgkin’s lymphoma, for which cyclophosphamide, doxorubicin, vincristine, and prednisone with rituximab(R–CHOP) is one of the standard regimens. Given that R–CHOP is highly emetogenic, chemotherapy-induced nausea and vomiting (CINV) prevention is clinically important. However, there is a paucity of studies focusing on these patients. This study aimed to ascertain the effectiveness of an oral fixed-dose combination of netupitant and palonosetron (NEPA) in preventing CINV in patients with DLBCL undergoing first-line R-CHOP chemotherapy. Seventy patients were enrolled in this single-center prospective non-comparative study conducted between November 2020 and May 2023 in South Korea. NEPA was administered 1 h prior to chemotherapy initiation on day 1. The primary endpoint of the study was the complete response rate (no emesis, and no rescue medication) during the acute, delayed, and overall phases, which were assessed over a period of 120 h post-chemotherapy. The complete response rates for NEPA were 90.0% [95% CI 80.5, 95.9] for the acute phase, 85.7% [95% CI 75.3, 92.9] for the delayed phase, and 84.3% [95% CI 73.6, 91.9] for the overall phase, with no-emesis rates (acute: 97.1% [95% CI 97.1, 99.7], delayed: 95.7% [95% CI 88.0, 99.1], overall: 92.9% [95% CI 84.1, 97.6]). NEPA was well tolerated with no severe treatment-emergent adverse events. NEPA exhibited substantial efficacy in mitigating CINV in DLBCL patients undergoing R–CHOP chemotherapy, demonstrating high CR and no-emesis rates, and favorable safety profiles.

## Introduction

Diffuse large B-cell lymphoma (DLBCL) is the most common type of non-Hodgkin’s lymphoma worldwide^1,2^, accounting for approximately 30–40% of non-Hodgkin’s lymphoma cases^3–5^. DLBCL is aggressive and necessitates prompt and comprehensive therapeutic intervention. However, with appropriate treatment, the 5 year overall survival rate for DLBCL has demonstrated a notable increase, reaching approximately 61.6%^6^. Therefore, due to its significant clinical implications, DLBCL warrants timely and radical management.

R–CHOP, a chemotherapy regimen consisting of rituximab, cyclophosphamide, doxorubicin, vincristine, and prednisone, is a standard regimen for newly diagnosed patients with DLBCL^7^. This multidrug regimen is classified as highly emetogenic, leading to chemotherapy-induced nausea and vomiting (CINV) as a significant adverse event^8–11^. CINV diminishes the patient’s quality of life and hampers oral intake and overall health status. Prophylactic antiemetic medication is recommended to mitigate CINV. Although corticosteroids have traditionally served as the cornerstone for antiemetic control, their efficacy as single agents is limited^12^. Furthermore, as the R-CHOP regimen already includes prednisolone, alternative agents are required. Newer antiemetics, particularly 5-HT3 receptor antagonists, have demonstrated remarkable effectiveness against acute emesis^13,14^. Moreover, NK1 receptor antagonists have proven useful in preventing both acute and delayed emesis^15^. Consequently, combining a corticosteroid, 5–HT3 receptor antagonist, and NK1 receptor antagonist has become the preferred regimen for preventing emesis in patients undergoing highly emetogenic chemotherapy^16–18^.

Among these drugs, an oral fixed-dose combination of netupitant and palonosetron (NEPA) has proven effective in preventing CINV during highly emetogenic chemotherapy^19^. NEPA comprises netupitant, a highly selective NK1 receptor antagonist, and palonosetron, a second-generation 5–HT3 receptor antagonist. Its dual coverage for acute and delayed emesis by targeting two critical emesis pathways has proven to be useful in many highly emetogenic chemotherapies^20^. However, in a pivotal study, the majority of enrolled patients had solid cancer primarily treated with intravenous chemotherapy^19^, and its efficacy was not specified in non-Hodgkin’s lymphoma patients treated with chemotherapy containing oral steroids. Subsequent published studies have similarly enrolled a small number of patients with lymphoma; hence, information is scarce^21–23^. This study aimed to assess the efficacy of NEPA in patients with DLBCL, the most common type of non-Hodgkin’s lymphoma, who received highly emetogenic R-CHOP as the first-line chemotherapy.

## Methods

### Study design and patients

This prospective, non-comparative study on the effectiveness of NEPA for the prevention of CINV was performed at a single center in South Korea. Seventy patients were enrolled between November 2020 and May 2023. Approval was obtained from the local institutional review board/ethics committee, and all patients provided written informed consent before enrollment in the study (IRB No. 2022AN0069). All research was performed in accordance with relevant guidelines/regulations.

Eligible patients were aged ≥ 19 years, histologically diagnosed with DLBCL undergoing R–CHOP as first-line chemotherapy with oral prednisolone included in the regimen. The exclusion criteria included patients with hypersensitivity to NEPA, pregnant or lactating women, patients with severe organ dysfunction, and patients with conditions that could cause nausea and vomiting, irrespective of chemotherapy.

### Treatment

The R–CHOP regimen consisted of rituximab (375 mg/m^2^ of body surface area (BSA) intravenously), cyclophosphamide (750 mg/ m^2^ of BSA intravenously), doxorubicin (50 mg/m^2^ of BSA intravenously), and vincristine (1.4 mg/ m^2^ of BSA intravenously; maximum dose: 2 mg) on day 1, and prednisolone (100 mg per day orally) on days 1–5. The physician determined dose reduction based on the patient’s general condition, eastern cooperative oncology group (ECOG) performance status, and underlying disease. Before rituximab administration, premedication consisting of intravenous paracetamol and pheniramine was administered. Patients were administered a fixed oral combination of netupitant (300 mg) and palonosetron 0.5 mg 1 h prior to chemotherapy initiation on day 1. In cases where patients experienced nausea or vomiting after the initiation of chemotherapy, rescue therapy was implemented using either oral or intravenous metoclopramide, if needed.

### Assessments

Nausea and vomiting were documented by patients in a study diary. Each emetic episode, use of rescue medication, and the maximum grade of nausea according to the numeric rating scale (NRS) were recorded daily from day 1 of chemotherapy until 120 h (day 5) after chemotherapy. Adverse events within 120 h of NEPA administration were recorded.

Safety was assessed by surveying every patient during chemotherapy and follow-up visits. The severity of CINV was graded according to the Common Toxicity Criteria for Adverse Events (CTCAE) version 4.0. The adverse events (AEs) were coded using the medical dictionary for regulatory activities, to provide the system organ class and preferred term for each event. Only treatment-emergent AEs (TEAEs) were considered.

The primary endpoint of the study was the complete response (CR) which was defined as no emetic episode and no rescue medication during the acute, delayed, and overall phases. Acute phase was defined as < 24 h after the chemotherapy infusion. The delayed phase was defined as > 24 h to 120 h post-chemotherapy. The overall phase was defined as the total period of 120 h post-chemotherapy. The secondary efficacy endpoints for this analysis included complete control (CR with a maximum nausea grade < 4), no emesis (regardless of rescue medication), no rescue use (regardless of emesis, patients could receive rescue medication only with nausea), and nausea severity during each phase. Nausea severity was classified as none, mild (NRS 1–3), moderate (NRS 4–6), and severe (7–10).

### Statistics

Mean and standard deviation were reported for continuous variables and percentage for categorical values. For each efficacy endpoint, the results were summarized as cumulative incidences and associated two-tailed 95% exact binomial confidence intervals (CIs). IBM SPSS version 27.0 (IBM Corp., Armonk, NY, USA) was used to analyze the data.

### Ethics approval

Approval was obtained from the Institutional Review Board (IRB) of Korea University Anam Hospital (IRB No. 2022AN0069).

## Results

### Baseline characteristics

Seventy patients with DLBCL were included in the analysis. The qualitative and quantitative demographic characteristics are summarized in Table 1. The median age of the patients was 66 years. Forty-three (61.4%) and 27 (38.6%) patients were male and female, respectively. Most patients had ECOG performance scores of 0 or 1 (87.1%).

**Table 1:** Summary of patient’s characteristics.

Median age, years (range)	66 (28–86)
Sex, *n* (%)	
Male	43 (61.4)
Female	27 (38.6)
Disease stage, *n* (%)	
Limited (stages I–II)	32 (45.7)
Advanced (stages III–IV)	38 (54.3)
Extranodal involvement, *n* (%)	54 (77.1)
Bone marrow involvement, *n* (%)^a^	7 (10)
B symptoms at baseline, n (%)	3 (4.3)
LDH above normal limit, *n* (%)	30 (43.5)
ECOG performance score, *n* (%)	
0–1	61 (87.1)
2–4	9 (12.9)
IPI score, *n* (%)	
< 3	37 (52.9)
≥ 3	33 (47.1)

^a^9 missing values.

*LDH* lactate dehydrogenase, *ECOG* eastern cooperative oncology group, *IPI* international prognostic index.

### Efficacy

**Figure 1:**
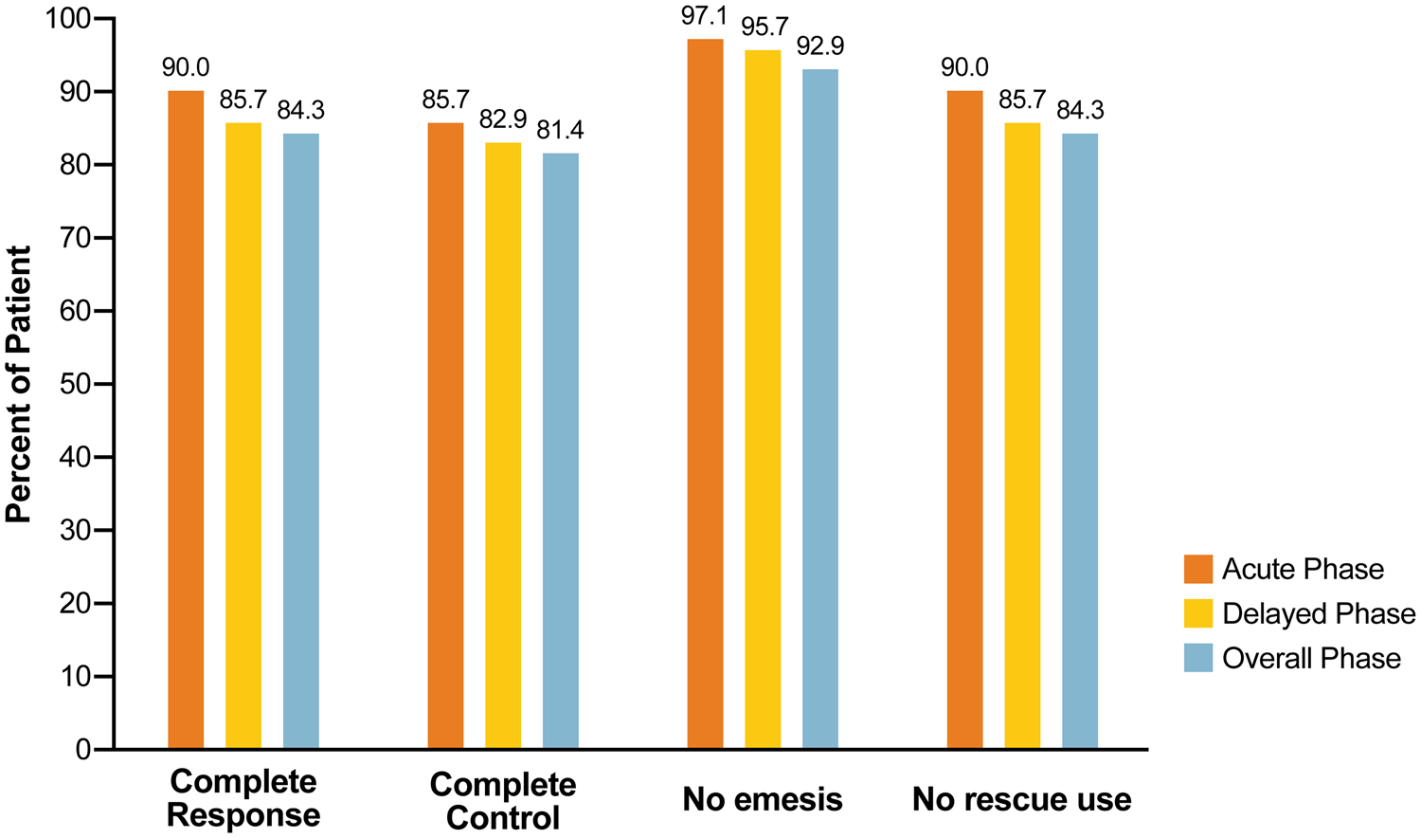
The histograms show the proportion of patients with complete response, complete control, no emesis, and no rescue medication use during the acute, delayed, and overall phases of chemotherapy.

NEPA demonstrated remarkable efficacy in preventing CINV in DLBCL patients undergoing R–CHOP chemotherapy. The CR rates for NEPA were 90.0% [95% CI 80.5, 95.9] for the acute phase, 85.7% [95% CI 75.3, 92.9] for the delayed phase, and 84.3% [95% CI 73.6, 91.9] for the overall phase (Fig. 1). Notably, NEPA exhibited exceptional effectiveness in achieving the endpoint of “no emesis,” with rates of 97.1% [95% CI 97.1, 99.7] for the acute phase, 95.7% [95% CI 88.0, 99.1] for the delayed phase, and 92.9% [95% CI 84.1, 97.6] for the overall phase.

**Figure 2:**
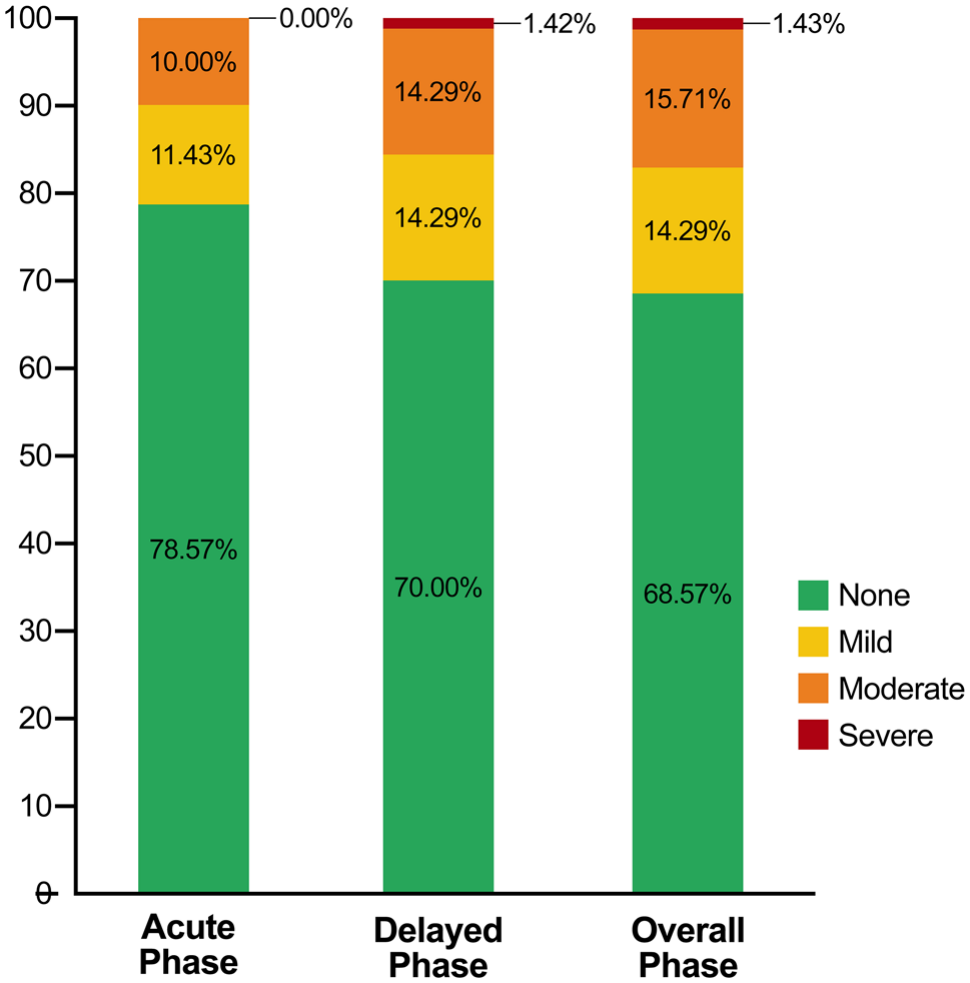
The histograms show the proportion of patients with no, mild, moderate, or severe nausea during the acute, delayed, and overall phases of chemotherapy.

The severity of nausea was also documented by patients. The findings revealed that most patients experienced either no nausea or mild nausea during the acute, delayed, and overall phases (Fig. 2). Specifically, 90% of the patients reported no nausea or mild nausea during the acute phase. In contrast, only one patient reported experiencing severe nausea during both the delayed and overall phases. However, this case was successfully resolved by the administration of rescue medications.

### Safety

The safety of NEPA was assessed in 70 patients. Only TEAEs were considered in this study. These AEs occurred after the first administration of the study medication. Overall, 77.1% of the patients experienced 109 TEAEs, but none experienced serious TEAEs. There was no definite association between the study drug and the observed TEAEs. One patient (1.4%) exhibited an allergic reaction, possibly due to the study medication. Constipation emerged as the most common adverse event, afflicting 38.6% of the patients, and was thought to be possibly related to NEPA. Other TEAEs include gastrointestinal symptoms such as indigestion and anorexia, neurological symptoms such as headache or insomnia, and generalized symptoms and signs such as edema or elevated serum chemistry levels. However, none of these events were evaluated as being possibly related to NEPA administration. The important TEAEs are summarized in Table 2. Overall, NEPA was well tolerated, and the severity of TEAEs was mild-to-moderate and resolved with adequate intervention.

**Table 2:** Treatment emergent adverse events^a^.

TEAEs	Number of patients (%)
Constipation^b^	27 (38.6)
Indigestion	7 (10)
Anorexia	5 (7)
Headache	4 (5.7)
Insomnia	4 (5.7)
Edema	4 (5.7)
Elevated serum creatinine	3 (4.3)
Elevated LFT	3 (4.3)
Epigastric pain	2 (2.8)
Anemia	2 (2.8)
Allergic reaction^b^	1 (1.4)
Tachycardia	1 (1.4)
Hypotension	1 (1.4)
Myalgia	1 (1.4)
Increased blood glucose	1 (1.4)
Dizziness	1 (1.4)
Hypomagnesemia	1 (1.4)
Hypokalemia	1 (1.4)
Diarrhea	1 (1.4)

^a^All TEAEs were mild to moderate. There were no severe TEAEs.

^b^Only constipation and allergic reaction was thought to be possibly related to the study drug. The rest of TEAEs are thought to be irrelevant.

*LFT* liver function test.

## Discussion

The efficacy of the administration of netupitant and palonosetron in the control of CINV has been previously demonstrated, mostly in solid cancer patients receiving highly emetogenic chemotherapy^19^. Our prospective study further substantiates its effectiveness in mitigating CINV in patients on R-CHOP chemotherapy. We observed CR rates of 90, 85.7, and 84.3% in the acute, delayed, and overall phases, respectively. Remarkably, 97.1% of the patients in the acute phase did not experience vomiting, underscoring the robust antiemetic properties of NEPA. None of the patients reported severe nausea in the acute phase, and only one patient reported severe nausea in the delayed phase.

As mentioned, the overall CR rate in our study was almost 84.3%, which is relatively high compared with other studies that investigated different antiemetic agents in similar patient populations receiving CHOP-based chemotherapy^24–27^. A literature review identified four studies on non-Hodgkin’s lymphoma patients receiving R–CHOP or CHOP chemotherapy. Of these, two were retrospective and two were prospective studies. Three of these studies assessed CR using the same criteria and timeframes as those used in our study. Takahashi et al. defined the overall phase as the period from the start of antiemetic administration (0 h) to 168 h afterwards^26^. While our study only included DLBCL patients undergoing R–CHOP chemotherapy, the patients in all four studies included not only DLBCL patients, but also other non-Hodgkin’s lymphoma patients undergoing CHOP chemotherapy with or without rituximab. Choi et al. also included the ProMACE-CytaBOM (cyclophosphamide, doxorubicin, etoposide, cytarabine, bleomycin, vincristine, methotrexate, leucovorin, and prednisone) regimen ^24^. Similar to our study, these four studies also administered antiemetics on the day 1 of the first chemotherapy cycle. CINV assessments were graded according to the CTCAE version 4.0. Only Choi et al. have assessed the safety of the antiemetic palonosetron in detail.

A retrospective study by Takahashi et al. on the efficacy of intravenous or oral 5–HT3 receptor antagonists reported an overall CR rate of 80.6% ^26^. Subgroup analysis showed no significant differences between the intravenous and oral agents. Choi et al. and Miyata et al. conducted prospective studies on the efficacy of palonosetron and showed overall CR rates of 68.2 and 70.0%, respectively^24,25^. Wakasugi et al. conducted a retrospective comparative analysis of granisetron combined with aprepitant and granisetron alone revealed CR rates of 80.0 and 83.3%, respectively. However, the difference between the groups was not statistically significant^27^. Compared with the above studies, the overall CR rate of 84.3% in our study seems relatively high, although a direct comparison cannot be made. Patients experienced constipation as the most common TEAE, and it was consistent with prior NEPA studies in other highly emetogenic chemotherapy^19,28^. Choi et al. also found that the most important drug related TEAEs were constipation and fatigue (2.3% each). Although these symptoms are generally mild and manageable with appropriate interventions, future studies should delve more deeply into this association. The results of each study are summarized in the Supplementary material.

One potential explanation for the observed higher control rate of CINV is the inclusion of high-dose corticosteroids in this regimen. Corticosteroids have a well-established safety and efficacy profile as monotherapy and in combination with other agents for the management of acute and delayed phase CINV^29^. In several controlled clinical trials, the combination of a 5–HT3 receptor antagonist and 20 mg of dexamethasone (equivalent to approximately 133 mg of prednisolone) has been demonstrated to be more effective in preventing CINV than 5–HT3 receptor antagonist monotherapy^30–32^. In the case of NK1 receptor antagonists, there are no direct comparisons between monotherapy and combination therapy with corticosteroids. However, it is known that the addition of high-dose corticosteroids is more effective in the management of CINV^33,34^. A prospective study of NEPA in breast cancer patients receiving chemotherapy regimens that included doxorubicin and cyclophosphamide, but not prednisone, demonstrated lower control rates of CINV compared to our study^35^. In the aforementioned study, patients exhibited complete response rates of 70.0, 85.7, and 60.0% during the acute, delayed, and overall phases, respectively. The regimen used included doxorubicin at 60 mg/m^2^ and cyclophosphamide at 600 mg/m^2^, while dexamethasone was administered at 12 mg on day 1 and 8 mg on days 2 to 3 for antiemetic purposes. Although direct comparisons are not feasible, these findings suggest that our chemotherapy regimen, which includes a higher corticosteroid than the routine dexamethasone doses for antiemesis, may contribute to the enhanced control rates of CINV.

Our study had several strengths. Being a prospective trial, our data on emesis are arguably more precise than those of retrospective studies that rely on medical records. Also, to the best of our knowledge, this is the first study to report the efficacy of NEPA specifically in patients with DLBCL receiving R-CHOP chemotherapy, especially given the inherent inclusion of corticosteroids in the regimen. According to the American society of clinical oncology, national comprehensive cancer network, and European society for medical oncology guidelines, a three-drug regimen including a 5–HT3 receptor antagonist, an NK1 receptor antagonist, and dexamethasone is recommended for preventing CINV in highly emetogenic chemotherapy, such as the CHOP regimen^8–10^. Our findings may pave the way for the routine incorporation of NEPA as a prophylactic measure, given its ease of administration and efficacy. Confirmation of the efficacy of oral NEPA is clinically significant because of the clinical convenience of switching from traditional intravenous antiemetics, which can be cumbersome for both healthcare providers and patients, to oral antiemetics.

This study had several limitations. First, we analyzed data from a small number of patients. Second, we did not directly compare NEPA with the control medication. We attempted to conduct a meta-analysis to compare our findings with those of other studies; however, this was not feasible due to the heterogeneity of the study designs. Instead, we present a review of the relevant literature. However, this does not adequately account for potential confounders that may influence CINV and should be interpreted cautiously. Finally, we did not survey patients’ drinking history, motion sickness, emesis during pregnancy, anxiety regarding chemotherapy, or other confounding factors that could be attributed to emetogenic susceptibility. Nonetheless, our results underscore robust efficacy of NEPA in CINV management, suggesting it is on par, if not superior, to other antiemetics.

In conclusion, our study emphasizes the strong efficacy of NEPA in preventing CINV in patients with DLBCL undergoing R–CHOP chemotherapy. With an impressive overall CR rate of 84.3% and minimal reports of severe nausea, NEPA demonstrated potent antiemetic potential. While our findings significantly enrich the existing literature, especially given the unique focus on patients with DLBCL, it is important to highlight the need for more detailed randomized clinical trials. These trials, which ideally compare NEPA with other agents, will further clarify its position in the field of antiemetics, ensuring optimal care for patients with DLBCL who are undergoing highly emetogenic chemotherapy.

## Consent to participate

All patients provided written informed consent before enrollment in the study.

### Supplementary Information

Supplementary Table 1.

## Data Availability

The datasets used and/or analysed during the current study is available from the corresponding author on reasonable request.

## References

[1] Perry AM, Diebold J, Nathwani BN, MacLennan KA, Müller-Hermelink HK, Bast M, Boilesen E, Armitage JO, Weisenburger DD (2016). Non-Hodgkin lymphoma in the developing world: Review of 4539 cases from the International non-hodgkin lymphoma classification project. Haematologica.

[2] Kanas G, Ge W, Quek RGW, Keeven K, Nersesyan K, Jon EA (2022). Epidemiology of diffuse large B-cell lymphoma (DLBCL) and follicular lymphoma (FL) in the United States and Western Europe: Population-level projections for 2020–2025. Leuk. Lymphoma.

[3] Teras LR, DeSantis CE, Cerhan JR, Morton LM, Jemal A, Flowers CR (2016). 2016 US lymphoid malignancy statistics by world health organization subtypes. CA Cancer J. Clin..

[4] Li XQ, Li GD, Gao ZF, Zhou XG, Zhu XZ (2012). Distribution pattern of lymphoma subtypes in China: A nationwide multicenter study of 10002 cases. J. Diagn. Concepts Pract..

[5] Kim JS, Liu Y, Ha KH, Qiu H, Rothwell LA, Kim HC (2020). Increasing incidence of B-cell non-hodgkin lymphoma and occurrence of second primary malignancies in South Korea: 10-year follow-up using the Korean national health information database. Cancer Res. Treat..

[6] Garg M, Takyar J, Dhawan A, Saggu G, Agrawal N, Hall A, Raut M, Ryland KE (2022). Diffuse large B-cell lymphoma (DLBCL): A structured literature review of the epidemiology, treatment guidelines, and real-world treatment patterns. Blood.

[7] NCCN (2023) *NCCN Clinical Practice Guidelines in Oncology (NCCN Guidelines®) B-Cell Lymphomas (Version 3. 2023)*. https://www.nccn.org/guidelines/guidelines-detail?category=1&id=1480. Accessed 1 June 2023.

[8] Hesketh PJ, Kris MG, Basch E (2020). Antiemetics: ASCO guideline update. J. Clin. Oncol..

[9] NCCN (2023) *NCCN Clinical Practice Guidelines in Oncology (NCCN Guidelines®) Antiemesis (Version 2.2023)*. https://www.nccn.org/guidelines/guidelines-detail?category=3&id=1415. Accessed 31 May 2023.

[10] Roila F, Molassiotis A, Herrstedt J (2016). 2016 MASCC and ESMO guideline update for the prevention of chemotherapy- and radiotherapy-induced nausea and vomiting and of nausea and vomiting in advanced cancer patients. Ann. Oncol..

[11] Coates A, Abraham S, Kaye SB, Sowerbutts T, Frewin C, Fox RM, Tattersall MH (1983). On the receiving end–patient perception of the side-effects of cancer chemotherapy. Eur. J. Cancer Clin. Oncol..

[12] Ioannidis JP, Hesketh PJ, Lau J (2000). Contribution of dexamethasone to control of chemotherapy-induced nausea and vomiting: a meta-analysis of randomized evidence. J. Clin. Oncol..

[13] Takahashi T, Okada T, Ikejiri F (2018). A prospective study of palonosetron for prevention of chemotherapy-induced nausea and vomiting in malignant lymphoma patients following highly emetogenic chemotherapy. Int. J. Clin. Oncol..

[14] Italian Group for Antiemetic Research (2000). Dexamethasone alone or in combination with ondansetron for the prevention of delayed nausea and vomiting induced by chemotherapy. N. Engl. J. Med..

[15] Morita M, Kishi S, Ookura M, Matsuda Y, Tai K, Yamauchi T, Ueda T (2017). Efficacy of aprepitant for CHOP chemotherapy-induced nausea, vomiting, and anorexia. Curr. Probl. Cancer.

[16] Gao HF, Liang Y, Zhou NN, Zhang DS, Wu HY (2013). Aprepitant plus palonosetron and dexamethasone for prevention of chemotherapy-induced nausea and vomiting in patients receiving multiple-day cisplatin chemotherapy. Intern. Med. J..

[17] Jordan K, Kinitz I, Voigt W, Behlendorf T, Wolf HH, Schmoll HJ (2009). Safety and efficacy of a triple antiemetic combination with the NK-1 antagonist aprepitant in highly and moderately emetogenic multiple-day chemotherapy. Eur. J. Cancer.

[18] Hamada S, Hinotsu S, Kawai K (2014). Antiemetic efficacy and safety of a combination of palonosetron, aprepitant, and dexamethasone in patients with testicular germ cell tumor receiving 5-day cisplatin-based combination chemotherapy. Support Care Cancer.

[19] Gralla RJ, Bosnjak SM, Hontsa A, Balser C, Rizzi G, Rossi G, Borroni ME, Jordan K (2014). A phase III study evaluating the safety and efficacy of NEPA, a fixed-dose combination of netupitant and palonosetron, for prevention of chemotherapy-induced nausea and vomiting over repeated cycles of chemotherapy. Ann. Oncol..

[20] Stathis M, Pietra C, Rojas C, Slusher BS (2012). Inhibition of substance P-mediated responses in NG108-15 cells by netupitant and palonosetron exhibit synergistic effects. Eur. J. Pharmacol..

[21] Aapro M, Jordan K, Scotté F, Celio L, Karthaus M, Roeland E (2022). Netupitant-palonosetron (NEPA) for preventing chemotherapy-induced nausea and vomiting: From clinical trials to daily practice. Curr. Cancer Drug Targ..

[22] Karthaus M, Oskay-Özcelik G, Wülfing P, Hielscher C, Guth D, Zahn MO, Flahaut E, Schilling J (2020). Real-world evidence of NEPA, netupitant-palonosetron, in chemotherapy-induced nausea and vomiting prevention: Effects on quality of life. Future Oncol..

[23] Aapro M, Rugo H, Rossi G (2014). A randomized phase III study evaluating the efficacy and safety of NEPA, a fixed-dose combination of netupitant and palonosetron, for prevention of chemotherapy-induced nausea and vomiting following moderately emetogenic chemotherapy. Ann. Oncol..

[24] Choi BS, Borsaru GP, Ballinari G, Voisin D, Di Renzo N (2014). Multicenter phase IV study of palonosetron in the prevention of chemotherapy-induced nausea and vomiting (CINV) in patients with non-hodgkin lymphomas undergoing repeated cycles of moderately emetogenic chemotherapy. Leuk. Lymphoma.

[25] Miyata Y, Yakushijin K, Inui Y (2016). A prospective study of the antiemetic effect of palonosetron in malignant lymphoma patients treated with the CHOP regimen. Int. J. Hematol..

[26] Takahashi T, Kumanomidou S, Takami S (2016). A retrospective study of R–CHOP/CHOP therapy-induced nausea and vomiting in non-Hodgkin’s lymphoma patients: A comparison of intravenous and oral 5-HT3 receptor antagonists. Int. J. Hematol..

[27] Wakasugi Y, Noda S, Ikuno Y, Horie M, Kito K, Minamiguchi H, Terada T (2019). Granisetron plus aprepitant versus granisetron in preventing nausea and vomiting during CHOP or R–CHOP regimen in malignant lymphoma: A retrospective study. J. Pharm. Health Care Sci..

[28] Zhang L, Lu S, Feng J, Dechaphunkul A, Chang J, Wang D, Chessari S, Lanzarotti C, Jordan K, Aapro M (2018). A randomized phase III study evaluating the efficacy of single-dose NEPA, a fixed antiemetic combination of netupitant and palonosetron, versus an aprepitant regimen for prevention of chemotherapy-induced nausea and vomiting (CINV) in patients receiving highly emetogenic chemotherapy (HEC). Ann. Oncol..

[29] Grunberg SM (2007). Antiemetic activity of corticosteroids in patients receiving cancer chemotherapy: Dosing, efficacy, and tolerability analysis. Ann. Oncol..

[30] Fauser AA, Pizzocaro G, Schueller J, Khayat D, Wilkinson P (2000). A double-blind, randomised, parallel study comparing intravenous dolasetron plus dexamethasone and intravenous dolasetron alone for the management of fractionated cisplatin-related nausea and vomiting. Support Care Cancer.

[31] Villalon A, Chan V (2004). Multicenter, randomized trial of ramosetron plus dexamethasone versus ramosetron alone in controlling cisplatin-induced emesis. Support Care Cancer.

[32] Italian Group for Antiemetic Research (1995). Dexamethasone, granisetron, or both for the prevention of nausea and vomiting during chemotherapy for cancer. N. Engl. J. Med..

[33] Watanabe D, Iihara H, Fujii H, Makiyama A, Nishida S, Suzuki A (2022). One-day versus three-day dexamethasone with nk1ra for patients receiving carboplatin and moderate emetogenic chemotherapy: A network meta-analysis. Oncologist.

[34] Minatogawa H, Izawa N, Shimomura K (2024). Dexamethasone-sparing on days 2–4 with combined palonosetron, neurokinin-1 receptor antagonist, and olanzapine in cisplatin: a randomized phase III trial (SPARED Trial). Br. J. Cancer.

[35] Yeo W, Lau TK, Kwok CC (2022). NEPA efficacy and tolerability during (neo)adjuvant breast cancer chemotherapy with cyclophosphamide and doxorubicin. BMJ Support Palliat. Care.

