# Supplementary material for: Efficacy and safety of netupitant/palonosetron in preventing nausea and vomiting in diffuse large B cell lymphoma patients undergoing R–CHOP chemotherapy
Source: Sci Rep. 2024 May 16;14:11229. doi: 10.1038/s41598-024-62057-4 (PMC11099181; doi:10.1038/s41598-024-62057-4)
Supplement: Supplementary file 1 — Supplementary Table 1. [file 41598_2024_62057_MOESM1_ESM.docx]

Title: Efficacy and Safety of Netupitant/Palonosetron in Preventing Nausea and Vomiting in Diffuse Large B Cell Lymphoma Patients Undergoing R-CHOP Chemotherapy

Journal name: Supportive Care in Cancer

Kunye Kwak^1^, Yong Park^1^, Byung Soo Kim^1^, Ka-Won Kang^1*^

^1^Division of Hematology-Oncology, Department of Internal Medicine, Korea University College of Medicine, Seoul, Republic of Korea

* E-mail: ggm1018@gmail.com

| Supplementary Table 1. Comparison of different studies regarding antiemetics in R-CHOP/CHOP therapy | | | | | | |
| --- | --- | --- | --- | --- | --- | --- |
|  | Our study | Study A^a^ | Study B^b^ | Study C^c^ | Study D^d^ | |
| No. of patients | 70 | 72 | 88 | 50 | 15 | 24 |
| Antiemetic  prophylaxis | Netupitant  Palonosetron | IV (granisetron) or  oral (ramosteron)  5-HT3 RA | Palonosetron | Palonosetron | Granisetron plus  aprepitant | Granisetron only |
| CR^e^ | Acute CR: 90.0% | Acute CR: 88.9% | Acute CR: 77.3% | Acute CR: 86% | Acute CR: 93.9% | Acute CR: 87.5% |
|  | Delayed CR: 85.7% | Delayed CR: 83.3% | Delayed CR: 83.3% | Delayed CR: 74% | Delayed CR: 80.0% | Delayed CR: 87.5% |
|  | Overall CR: 84.3 % | Overall CR: 80.6 % | Overall CR: 68.2% | Overall CR: 70% | Overall CR: 80.0% | Overall CR: 83.3% |
|  |  | No significant difference in CR rate between patients who were treated with oral and IV agents |  |  | No significant difference in CR rate between the two groups | |

Abbreviations: R-CHOP, rituximab with cyclophosphamide, doxorubicin, vincristine, and prednisone; CR, complete response; IV, intravenous; 5-HT3 RA, serotonin receptor antagonists

^a^Study A: Takahashi T, Kumanomidou S, Takami S, et al. (2016) A retrospective study of R-CHOP/CHOP therapy-induced nausea and vomiting in non-Hodgkin’s lymphoma patients: a comparison of intravenous and oral 5-HT3 receptor antagonists. Int J Hematol 104(3):378–383. https://doi.org/10.1007/s12185-016-2041-z

^b^Study B: Choi BS, Borsaru GP, Ballinari G, Voisin D, Di Renzo N (2014) Multicenter phase IV study of palonosetron in the prevention of chemotherapy-induced nausea and vomiting (CINV) in patients with non-Hodgkin lymphomas undergoing repeated cycles of moderately emetogenic chemotherapy. Leuk Lymphoma 55(3):544–550. <https://doi.org/10.3109/10428194.2013.813498>

^c^Study C: Miyata Y, Yakushijin K, Inui Y, et al. (2016) A prospective study of the antiemetic effect of palonosetron in malignant lymphoma patients treated with the CHOP regimen. Int J Hematol 104(6):682–691. <https://doi.org/10.1007/s12185-016-2089-9>

^d^Study D: Wakasugi Y, Noda S, Ikuno Y, et al. (2019) Granisetron plus aprepitant versus granisetron in preventing nausea and vomiting during CHOP or R-CHOP regimen in malignant lymphoma: a retrospective study. J Pharm Health Care Sci 5:24. <https://doi.org/10.1186/s40780-019-0153-3>

^e^Study A (Takahashi et al.) defined the overall phase as the period from the start of antiemetic administration (0 h) to 168 h afterwards. Study B, C and D assessed overall phase until 120h, using the same timeframes as those used in our study. Study A, B, C and D had the same CR definition which was defined as no emetic episode and no rescue medication.
